# NCAM and attached polysialic acid affect behaviors of breast epithelial cells through differential signaling pathways: Polysialylation in regulating NCAM function

**DOI:** 10.3724/abbs.2024176

**Published:** 2024-10-15

**Authors:** Yurong Wu, Juhong Yang, Xin Wang, Jia Guo, Zengqi Tan, Feng Guan, Lin Cao

**Affiliations:** 1 Provincial Key Laboratory of Biotechnology Joint International Research Laboratory of Glycobiology and Medicinal Chemistry College of Life Sciences Northwest University Xi’an 710069 China; 2 Shandong University of Arts Ji’nan 250300 China; 3 Institute of Biomedical Engineering and Health Sciences Changzhou University Changzhou 213164 China; 4 Provincial Key Laboratory of Biotechnology Joint International Research Laboratory of Glycobiology and Medicinal Chemistry School of Medicine Northwest University Xi’an 710069 China

**Keywords:** neural cell adhesion molecule, polysialic acid, epithelial-mesenchymal transition, β-catenin/slug, EGFR/STAT3

## Abstract

Neural cell adhesion molecule (NCAM), a common mammalian cell surface glycoprotein, is the major substrate of polysialic acid (polySia). Polysialylated NCAM occurs in many types of cancer, but rarely in normal adult tissues. The functional role of NCAM hypersialylation in the epithelial-mesenchymal transition (EMT) process remains unclear. The present study indicates that NCAM and attached polysialic acid affect behaviors of breast epithelial cells through differential signaling pathways. NCAM and polysialylated NCAM are aberrantly regulated in breast cancer cells. They are both upregulated in normal breast epithelial cells undergoing EMT. Western blot analysis demonstrates that NCAM-140 overexpression induces EMT in breast epithelial cells and promotes cell proliferation and migration through activation of the β-catenin/slug signaling pathway. Modification of polySia attached to NCAM modulates cell adhesion and promotes cell motility through activation of the EGFR/STAT3 pathway. These observations contribute to clarifying the molecular mechanisms by which polysialic acid and its major substrate, NCAM, modulate cell behaviors, and highlight the significance of increased polysialylated expression on NCAM during EMT and tumor development.

## Introduction

Sialic acid is a vital monosaccharide often found at the terminal position of cell-surface glycan chains [1]. Elevated expression of sialylation is correlated with tumor aggressiveness, invasion, drug resistance and poor prognosis in cancer patients [ 2, 3]. Aberrant sialylation is primarily due to abnormal regulation of sialyltransferases, the enzymes that catalyze the linkage of sialic acid to other carbohydrates [ 4, 5]. For example, overexpression of ST3GalI in breast cancer cells promotes tumorigenesis in a murine model [6], and upregulation of ST

6GalI is essential for the maintenance of cancer cells stemness [7]. Polysialyltransferases II (ST8SiaII) and IV (ST8SiaIV) transfer sialic acid from CMP-sialic acid to sialic acid residues of other sialoglycans to yield 2,8-linked polymerized structures (polySia) [8]. PolySia is associated with tumor development, including glioblastoma [9], lung cancer [10], and many other cancers [ 11, 12]. It has been reported that polySia is expressed in breast cancer MCF7 cells [13], but few studies have focused on the biological role of polySia in breast cancer progression. Neural cell adhesion molecule (NCAM) is the major polySia substrate in cancer cells [14]. Polysialylated NCAM inhibits cell-cell/cell-ECM interactions due to the steric effects of polySia. Therefore, polySia-NCAM is used as diagnostic marker because of the highly invasive and proliferative characteristics in polySia-expressing cancers [15].

NCAM is a member of the immunoglobulin superfamily cell adhesion molecules and has three isoforms (NCAM-120, NCAM-140, NCAM-180) based on alternative splicing [16]. NCAM-140 and -180 are transmembrane proteins; NCAM-120 is a glycosylphosphatidylinositol (GPI)-anchored protein. The extracellular region of NCAM comprises five immunoglobulin-like (Ig1-5) domains with six N-glycosylation sites and two fibronectin type-III-like (FN1-2) domains [15]. Elevated NCAM expression has been reported in pancreatic cancer [17], neuroblastoma [18], small cell lung cancer [19], and other cancers. Polysialylated NCAM levels are also correlated with tumor metastasis [20]. NCAM plays critical roles as a signal transducer in regulating cell migration proliferation, apoptosis and EMT in multiple cancer cells [21], but polySia has not been specifically evaluated in that context. The effects of hypersialylation or hyposialylation of NCAM on various cell behaviors remain unclear.

In this study, breast epithelial cell lines were used to investigate the role of polySia and its major substrate, NCAM, in modulating various cell biological behaviors during EMT, revealing the regulatory role of polySia modification on the function of NCAM.

## Materials and Methods

### Antibodies and reagents

Antibodies used were mouse anti-E-cad IgG2a (part #610181), mouse anti-β-catenin IgG1 (610153), mouse anti-CD56 (NCAM13; 556324), anti-CD56 (NCAM 12F8; 556325), anti-STAT3 (610189) from BD Biosciences (San Jose, USA); mouse anti-N-cad IgG1 (sc59987), anti-CK1α (sc6477), anti-c-Myc (sc40) from Santa Cruz Biotechnology (Santa Cruz, USA); mouse anti-VM IgG1 (V5255), mouse anti-polysialylated NCAM antibody IgM 5A5 from Developmental Studies Hybridoma Bank (University of Iowa, Iowa City, USA); anti-β-tubulin I IgG1 (T7816), anti-FN (F3648) from Sigma-Aldrich (St Louis, USA); anti-EGFR (D38B1), anti-p-EGFR (Tyr 1068; D7A5), anti-p-STAT3 (Tyr 705; D3A7), anti-GSK-3β (27C10), anti-slug (C19G7), anti-histone H3 (D1H2) from Cell Signaling Technology (Beverly, USA); horseradish peroxidase (HRP)-labeled goat anti-mouse IgG (A0216), HRP-labeled goat anti-rabbit IgG (A0208), FITC-labeled goat anti-mouse IgG (A0568), Cy3-labeled goat anti-mouse IgG (A0521), donkey anti-goat IgG (A0181), anti-His-tag mAb (AH367) from Beyotime Institute of Biotechnology (Haimen, China); FITC-labeled goat anti-rabbit IgG (CW0114), goat anti-rat IgG (CW0166) from CWBIO (Beijing, China). FN, laminin, collagen IV, puromycin, and hygromycin were from Sigma-Aldrich. Matrigel was from Corning Life Sciences (Tewksbury, USA). Other reagents were from Sigma-Aldrich unless described otherwise.

### Cell lines and cell culture

Mouse mammary epithelial cell line NMuMG and human breast epithelial cell line MCF10A were from American Type Culture Collection (ATCC, Manassas, USA). Cells were cultured in DMEM (Hyclone, Logan, USA) supplemented with 10% FBS (Hyclone), 100 IU/mL penicillin, and 100 μg/mL streptomycin (Gibco, Carlsbad, USA) in a humidified 5% CO _2_ atmosphere at 37°C. For NMuMG, medium was supplemented with 10 μg/mL insulin (Sigma-Aldrich). For MCF10A, medium was supplemented with 1% sodium pyruvate (Solarbio, Beijing, China).

### Patients and tissue samples

Breast cancer (BC) tissues and normal breast tissues were obtained from the First Affiliated Hospital of Xi’an Jiaotong University (Xi’an, China). Informed consent was obtained from all patients in accordance with the Declaration of Helsinki. Experiments using human tissues were approved by the Research Ethics Committee of Northwest University.

### Plasmid construction and transfection

Mouse genes NCAM-120, NCAM-140, and ST8SiaII were amplified by PCR as previously described [22] using primers listed in Table 1. Then, PCR products were ligated into vector pcDNA3.1 (Invitrogen, Carlsbad, USA) or pIREShyg3 (Clontech, Mountain View, USA), respectively. NMuMG cells were stably transfected with polyethylenimine MAX (PEI; Polysciences Inc, Warrington, USA) and selected with G418 or hygromycin.

**Table:** **Table 1** The primers of mouse gene used in PCR

Gene	Primer sequence
NCAM-120	Sense	5′-CCCAAGCTTGCCACCATGCTGCGAACTAAGGATC-3′
	Antisense	5′-CCGCTCGAGTCAGAGCAGAAGAAGAGTCAC-3′
NCAM-140	Sense	5′-CCCAAGCTTGCCACCATGCTGCGAACTAAGGATC-3′
	Antisense	5′-CCGCTCGAGCGGTCATGCTTTGCTCTCATTC-3′
ST8SiaII	Sense	5′-GAAGGCCTGCCACCATGCAGCTGCAGTTCCG-3′
	Antisense (containing the codons for 6His residues)	5′-CTAGCTAGCTTAGTGGTGGTGGTGGTGGTGCGTAGC CCCATCACACT-3′

### Semi-quantitative PCR and quantitative real-time PCR

Semi-quantitative PCR and quantitative real-time PCR was performed as previously described [22]. Briefly, total RNA was isolated using an RNApure Tissue Kit (CWBIO) and reverse-transcripted to cDNA using a ReverTra Ace-α Kit (Toyobo, Osaka, Japan). Primers were designed as shown in Supplementary Table S1. Quantitative real-time PCR (RT-qPCR) was performed with UltraSYBR Mixture (CWBIO) using a CFX96 PCR detection system (Bio-Rad, Hercules, USA). Gene expression was quantified by the 2 ^‒ΔΔCt^ method [23] and expressed as the mean±SD from triplicate experiments.

### Migration assay

Migration assay was performed as previously described [22]. Briefly, cells (5×10 ^4^) were plated in an upper transwell insert without coating matrix (8-μm pore size; Corning Life Sciences) in serum-free medium, and complete-medium was added to the bottom chamber. After 24 h of culture, cells migrated across the membrane were stained with 0.1% crystal violet, and photographed under a microscope (Sunny Optical Technology, Hangzhou, China) at a magnification of 100×.

### Wound healing assay

NMuMG cells were plated at a high density in 6-well plates and incubated until a confluent monolayer was achieved. A scratch was made with 100-μL pipette tips in each well and cultures were washed with PBS to remove any cell debris. Cells were incubated in DMEM supplemented with 10% FBS and 5 μg/mL mitomycin C (Sigma-Aldrich) for 24 h. Cell migration between the scratch areas was monitored at 0 and 24 h using an optical microscope. Migration distance was measured with Image Pro Plus 6.0 (Media Cybemetics, Silver Spring, USA)

### MTT assay

Cell proliferation was determined by MTT assay as described previously [24]. Briefly, cells (4×10 ^3^/well) in 96-well plates were incubated 4 h with 4 μL MTT solution (Cers, Yantai, China). The reaction was terminated by addition of 100 μL DMSO, and absorbance at 595 nm was determined.

### Cell motility assay

Cell motility was determined by phagokinetic gold sol assay as described previously [25]. Cells (2×10 ^3^) in complete culture medium were seeded onto gold sol-coated wells, incubated for 12‒18 h, and photographed under an inverted microscope. Tracking areas of 50 cells were measured using the ToupView imaging system (Jingtong Instrument, Suzhou, China) and expressed as square pixels.

### Cell adhesion assay

Adhesion assays were performed as described previously [26]. In brief, 96-well plates were coated overnight at 37°C with FN (1 μg/well), collagen IV (1.5 μg/well), Matrigel (80 μg/well), or laminin (1 μg/well). Wells were rinsed and blocked for 1 h with 1% BSA in Hank’s balanced salt solution (HBSS) at 37°C. Cells were harvested with trypsin, plated (40,000 cells per coated well), and incubated 30 min at 37°C. Wells were washed gently with HBSS to remove unattached cells. Adherent cells were fixed with 4% paraformaldehyde for 10 min, stained with 0.1% crystal violet (in 20% methanol) for 10 min, dissolved in 100 μL of 10% acetic acid after removal of excess dye with PBS, and absorbance was measured at 595 nm.

### Immunofluorescence staining assay

Immunofluorescence staining was performed as previously described [22]. Cells (2×10 ^4^) coated on glass cover slips in 24-well plates, were washed with PBS, fixed with 4% fresh paraformaldehyde, blocked with 1% BSA, incubated with appropriate antibody and Hoechst 33342 (Invitrogen), mounted with Glycergel (Dako, Carpinteria, USA), and observed with a fluorescence microscope (Eclipse Ti-U; Nikon, Tokyo, Japan) at 600× magnification.

### Western blot analysis

Western blot analysis was performed as previously described [22]. Equal amounts of proteins were loaded on SDS-PAGE gels and transferred onto a PVDF membrane. The membrane was blocked with 5% BSA, incubated with primary antibody and appropriate HRP-conjugated secondary antibody, visualized by Pro-Light HRP (Tiangen Biotech, Beijing, China), and photographed using a Molecular Imager ChemiDoc XRS+ system (Bio-Rad).

### Flow cytometry assay

Cells were plated in triplicate in 24-well plates (2×10 ^5^ cells/well) as described previously [22], detached, incubated with primary and secondary FITC-conjugated antibodies. Signals from cells were detected by flow cytometry using a FACSCalibur flow cytometer (BD, San Jose, USA), with data acquisition and analysis by the FlowJo software program (Tree Star, San Carlos, USA).

### Gene silencing with small interfering RNA (siRNA)

Duplexes of 21 nucleotides of mouse ST8SiaII siRNA target sequence and negative control siRNA (NC), having no homology to other known mouse genes, were designed and synthesized by Invitrogen. The sequence for mouse ST8SiaII siRNA is 5′-GCCUGGAGAUAUUAUUCAUTT-3′ and the sequence for negative control siRNA is 5′-TTCTCCGAACGTGTCACGT-3′. SiRNA was transfected using Lipofectamine 2000 reagent, and cells were examined after 24 h. Suppression of ST8SiaII expression was verified by semi-quantitative and quantitative RT-PCR.

### Luciferase reporter assay

β-Catenin transcription was assessed using TOP FLASH/ FOP FLASH reporter luciferase assay [27]. Cells were seeded into 24-well plates and transfected using Lipofectamine 2000 (Invitrogen) with 1 μg M50 Super 8x TOP Flash (Plasmid 12456) or M51 Super 8x FOP Flash (Plasmid 12457) reporter vector (Addgene, Cambridge, USA), together with 0.05 μg internal pRL-TK Renilla plasmid (Promega, Madison, USA). Cells were processed 48 h after co-transfection for luciferase reporter activity using a Dual Luciferase Reporter System (Promega). Firefly luciferase activity was normalized against Renilla luciferase activity. Reporter assay results were presented as the relative luciferase activity (averaged ratio of firefly/Renilla luciferase±SE) from three or more independent experiments.

### Immunohistochemistry

Tissue slides were dewaxed and rehydrated. After antigen retrieval, slides were incubated with 3% hydrogen peroxide for 30 min and blocked in 10% normal mouse serum for 30 min. The slides were then incubated with primary antibodies against NCAM (1:1000; Santa Cruz) at 4°C overnight. The slides were rinsed with PBS, incubated with HRP-conjugated secondary antibody, visualized with DAB (Sigma-Aldrich). The integrated optical density (IOD) of the NCAM and the tissue area were measured using Image-Pro Plus software (version 6.0; Media Control Sciences, Rockville, USA). The NCAM expression intensity was expressed as the IOD per unit area.

### Statistical analysis

Data were statistically analyzed using the GraphPad Prism (GraphPad software; San Diego, USA). Differences between means were evaluated by Student’s *t*-test, and *P*<0.05 was considered significant.

## Results

### Expression of NCAM in clinical BC samples

**Figure 1:**
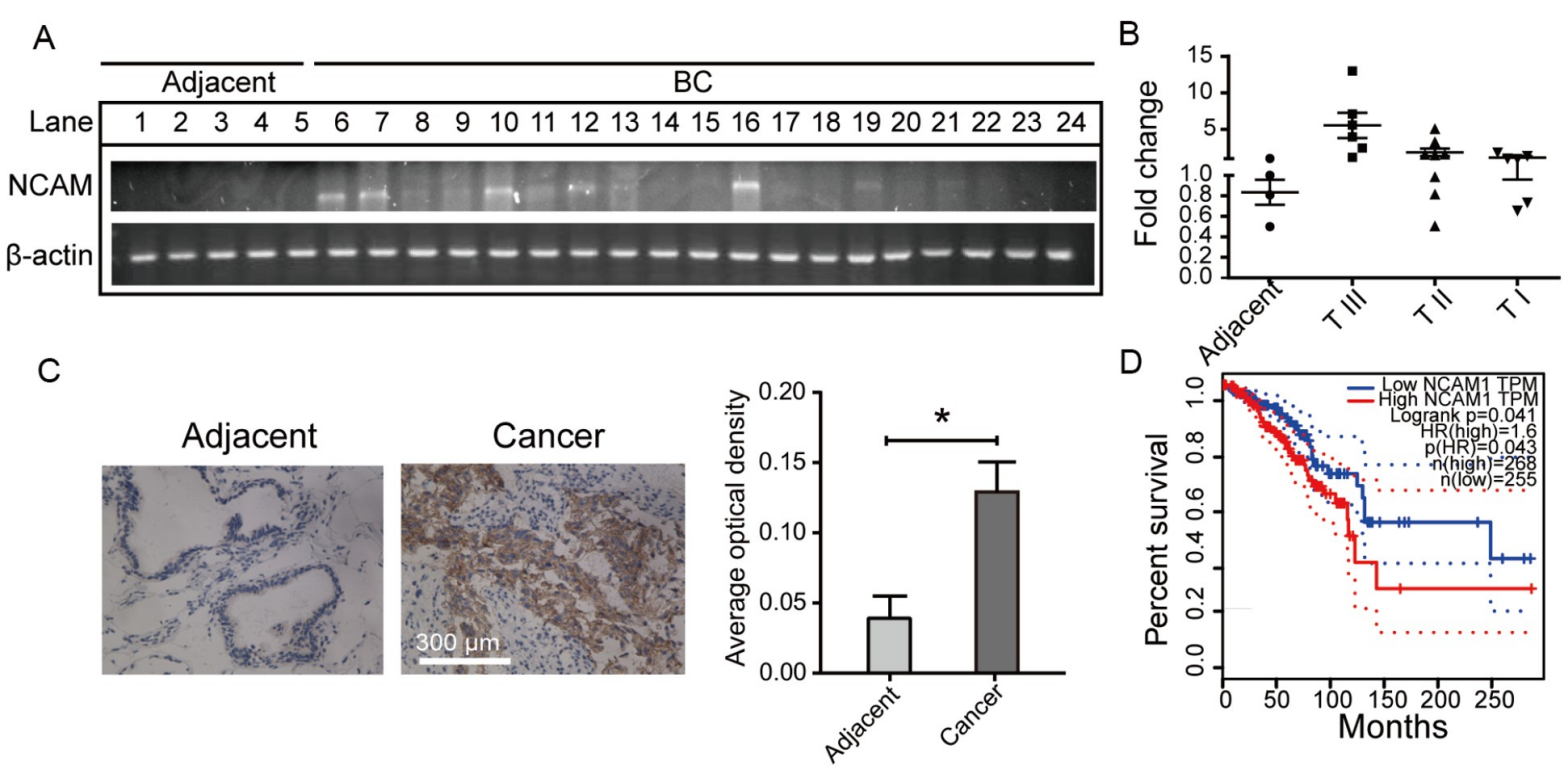
Figure 1 Expression of NCAM in human BC samples (A,B) mRNA levels of NCAM in malignant tissues (n=20) were compared with those in adjacent non-cancerous tissues (n=4) by PCR (A) and RT-qPCR (B). β-actin: loading control. Lanes 1‒4: adjacent non-cancerous tissues. Lanes 5‒10: BC tissues from TNM III patients. Lanes 11‒18: BC tissues from TNM II patients. Lanes 19‒24: BC tissues from TNM I patients. (C) Immunohistochemical staining of NCAM in BC samples. Malignant tissues and matching adjacent non-cancerous tissues were obtained from BC patients. One typical sample pair is shown (C, left), histogram is shown (C, right). Scale bars: 300 μm. *P<0.05. (D) Kaplan-Meier overall survival (OS) curves according to NCAM expression levels analyzed using the GEPIA tool (http://gepia.cancer-pku.cn). Results are shown as the mean±SD from triplicate experiments.

In the previous study, we observed significantly increased polySia and ST8SiaIV levels in BC samples compared to those in adjacent non-cancerous tissues [28]. Since NCAM is the major polySia substrate [29], NCAM expression at the mRNA level was evaluated by semi-quantitative PCR ( Figure 1A) and RT-qPCR ( Figure 1B) in 24 clinical BC tissue samples in this study. NCAM expression was higher in BC tissues than in adjacent non-cancerous tissues ( Figure 1A,B and Supplementary Tables S2, S3). Immunohistochemical staining showed positive NCAM signals in BC tissues but not in adjacent non-cancerous tissues ( Figure 1C). In Kaplan-Meier survival estimate, the mean survival time of the BC patients with high expression of NCAM was shorter than that of those with low expression of NCAM ( Figure 1D), suggesting that NCAM plays an essential role in BC tumorigenesis and progression.

### Aberrant regulation of NCAM-140 and polysialylated NCAM during EMT

**Figure 2:**
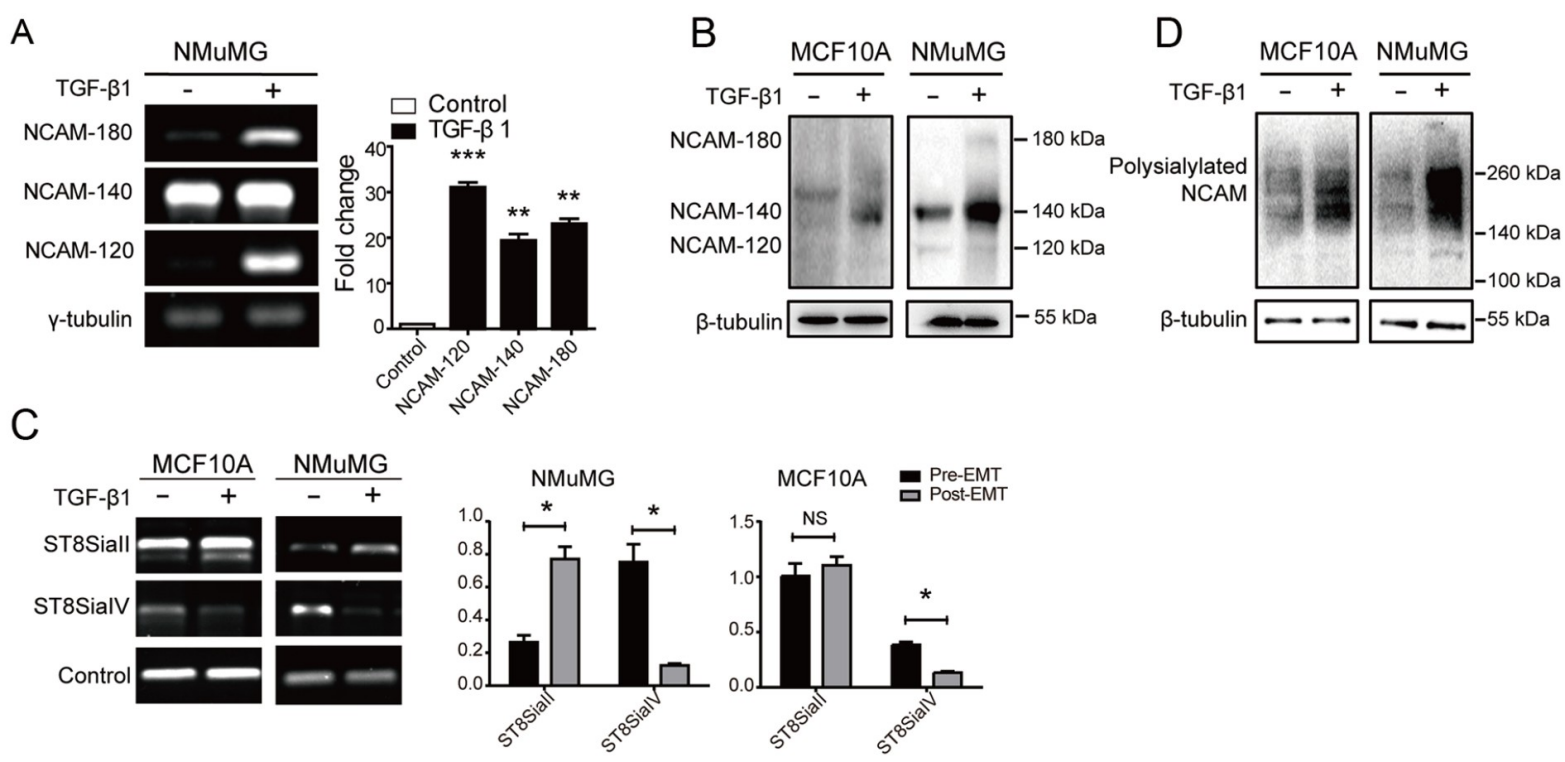
Figure 2 Altered NCAM-140 and polysialylated NCAM expression during EMT (A) mRNA levels of three NCAM isoforms in NMuMG cells during EMT. Semi-quantitative PCR (left panel) and RT-qPCR (right panel) were performed. γ-tubulin: loading control. **P<0.01, ***P<0.001. (B) Western blot analysis of NCAM in control and TGF-β1-treated MCF10A and NMuMG cells. (C) Expressions of ST8SiaII and ST8SiaIV at the mRNA level in MCF10A and NMuMG cells during EMT. Cells were treated (+) or not (‒) with TGF-β1 (5 ng/mL) for 48 h, and semiquantitative PCR was performed. The loading controls in left panel: β-actin, and in right panel: γ-tubulin. *P<0.05. (D) Western blot analysis of polysialylated NCAM during EMT. Data are shown as the mean±SD from three independent experiments.

EMT is a basic and highly conserved process that plays crucial roles in embryogenesis, cancer invasion and metastasis [24]. Expression of NCAM and polysialylated NCAM was studied in an *in vitro* EMT model established by TGF-β1 induction in MCF10A and NMuMG cells. Changes in protein levels associated with EMT were observed, including increase of N-cadherin (N-cad) (“cadherin switch”), and decreases of E-cadherin (E-cad), tumor markers vimentin (VM) and fibronectin (FN) ( Supplementary Figure S1A). The mRNA levels of various NCAM isotypes were elevated in NMuMG cells undergoing EMT ( Figure 2A). The protein levels of NCAM-140, but not NCAM-120 or NCAM-180, were upregulated in MCF10A and NMuMG cells undergoing EMT ( Figure 2B). It is known that NCAM can be polysialylated by the polysialyltransferases ST8SiaII and ST8SiaIV. Therefore, we analyzed ST8SiaII and ST8SiaIV expressions in the two model cell lines. Increased ST8SiaII expression and reduced ST8SiaIV expression were observed at the mRNA level during EMT ( Figure 2C). Polysialylated NCAM expression was much higher in cells undergoing EMT ( Figure 2D), indicating that upregulation of NCAM-140 and polysialylated NCAM is involved in the EMT process.

### NCAM-140 overexpression induces EMT in NMuMG cells

**Figure 3:**
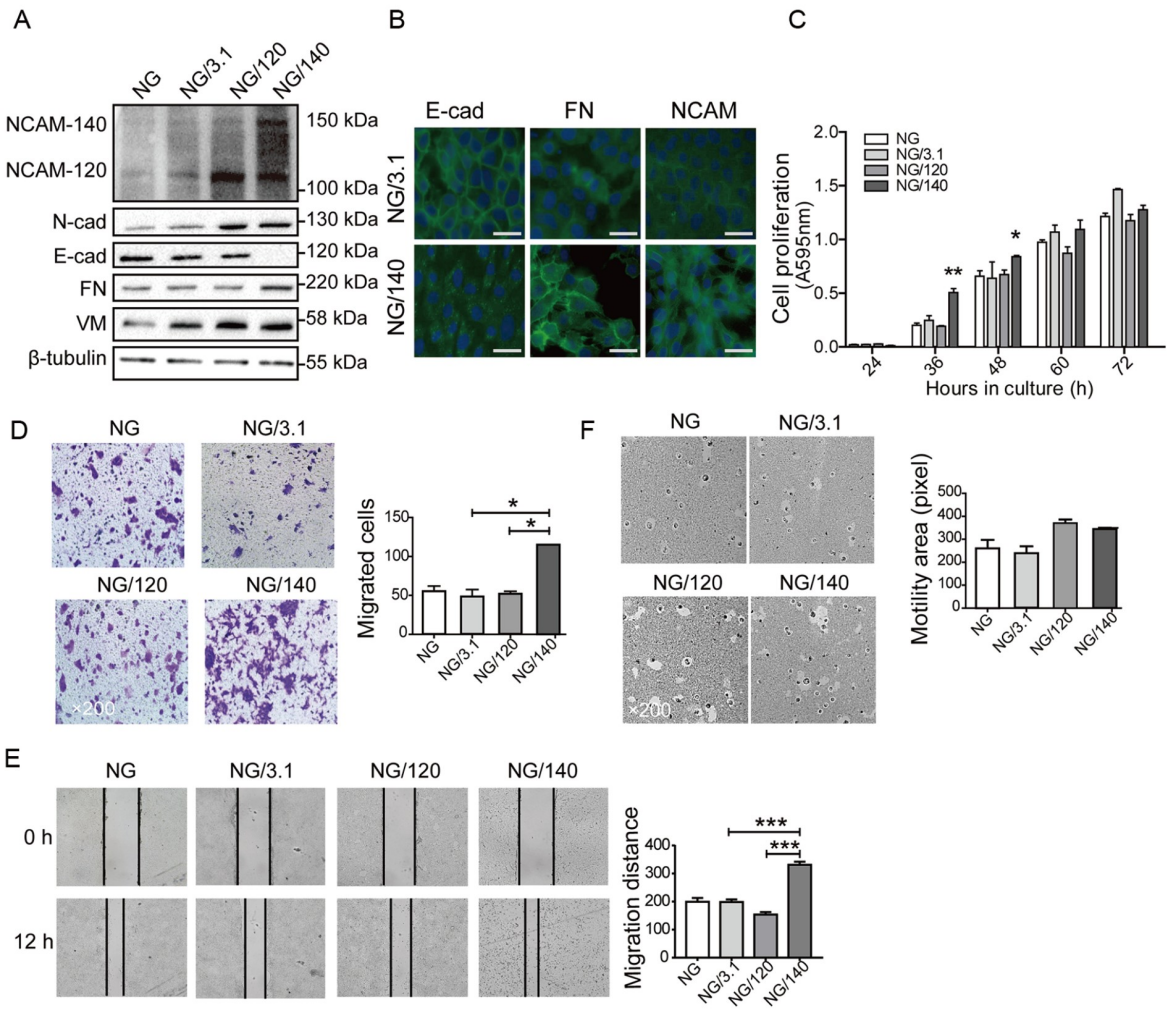
Figure 3 Differential effects of NCAM-120 and NCAM-140 on cell behaviors (A) Western blot analysis of EMT markers in NCAM-overexpressing NMuMG cells. β-tubulin: loading control. (B) Immunofluorescence staining of E-cad, FN, and NCAM in NCAM-140-overexpressing cells. NG/3.1 and NG/140 cells were cultured, and the nuclei were visualized by Hoechst staining. Scale bar: 20 μm. (C) Proliferation assay. Transfected cells were cultured for 24, 36, 48, 60, or 72 h and proliferation was assessed by MTT assay. *P<0.05, **P<0.01. (D) Migration assay. Cells were cultured for 48 h as described above, and migration was assessed as described in Materials and Methods. Migrating cells were quantified, and values are shown as the mean±SD. Two independent experiments gave similar results. Magnification: 200×. *P<0.05. (E) Wound healing assay, performed as described in Materials and Methods. Confluent cells were wounded, incubated with 5 μg/mL mitomycin for 24 h, and wounds were photographed and marked using ImagePro Plus software. Results are presented as the average of migration distance (0‒24 h)±SD from three independent experiments. ***P<0.001. (F) Motility assay, performed as described in Materials and Methods. Cleared areas on gold sol were measured as square pixels using the ToupView Image program. NG: NMuMG cells; NG/120: NMuMG-120 overexpressing cells; NG/140: NMuMG-140 overexpressing cells; NG/3.1: NMuMG cells transfected with vector pcDNA3.1. Data are shown as the mean±SD from three independent experiments.

To evaluate the effects of NCAM isoforms on cell behavior, we cloned the genes encoding NCAM-120 and NCAM-140, and transfected them separately into NMuMG cells. In contrast to a previous finding that ectopic expression of NCAM caused cell death of NMuMG [30], we obtained stable transfectants of the two isoforms, termed NG/120 and NG/140 cells ( Supplementary Figure S1B and Figure 3A). NG/140 had motile mesenchymal cell morphology, whereas NG/120 retained epithelial morphology similar to that of non-transfected cells (“NG”) ( Supplementary Figure S1C). N-cad and VM were upregulated in NG/120 and NG/140, but not in NG. Compared to NG or NG/3.1 (transfected with vector pcDNA3.1), NG/140 but not NG/120 showed nearly complete loss of E-cad expression, significantly enhanced FN expression ( Figure 3A,B), increased cell proliferation and migration abilities ( Figure 3C‒E). However, the motility of NG/140 was similar to the other NG cell lines ( Figure 3F). These findings suggest that NCAM-140 overexpression switches cells to an EMT-like process, with consequent alteration of proliferation and migration ability.

The mRNA level of NCAM-180 was obviously upregulated undergoing EMT in MCF10A and NMuMG cells, but the protein levels of NCAM-180 and NCAM-120 have not changed. Cell migration and proliferation abilities of NMuMG cells are mainly affected by the overexpression of NCAM-140, but not by NCAM-120. Of these three isoforms, only NCAM-140 was differentially expressed in the EMT process, and played an essential role in cell migration and proliferation. Consequently, the subsequent research primarily focused on NCAM-140.

### Differential effects of polysialylated NCAM and NCAM-140 on cell behaviors

**Figure 4:**
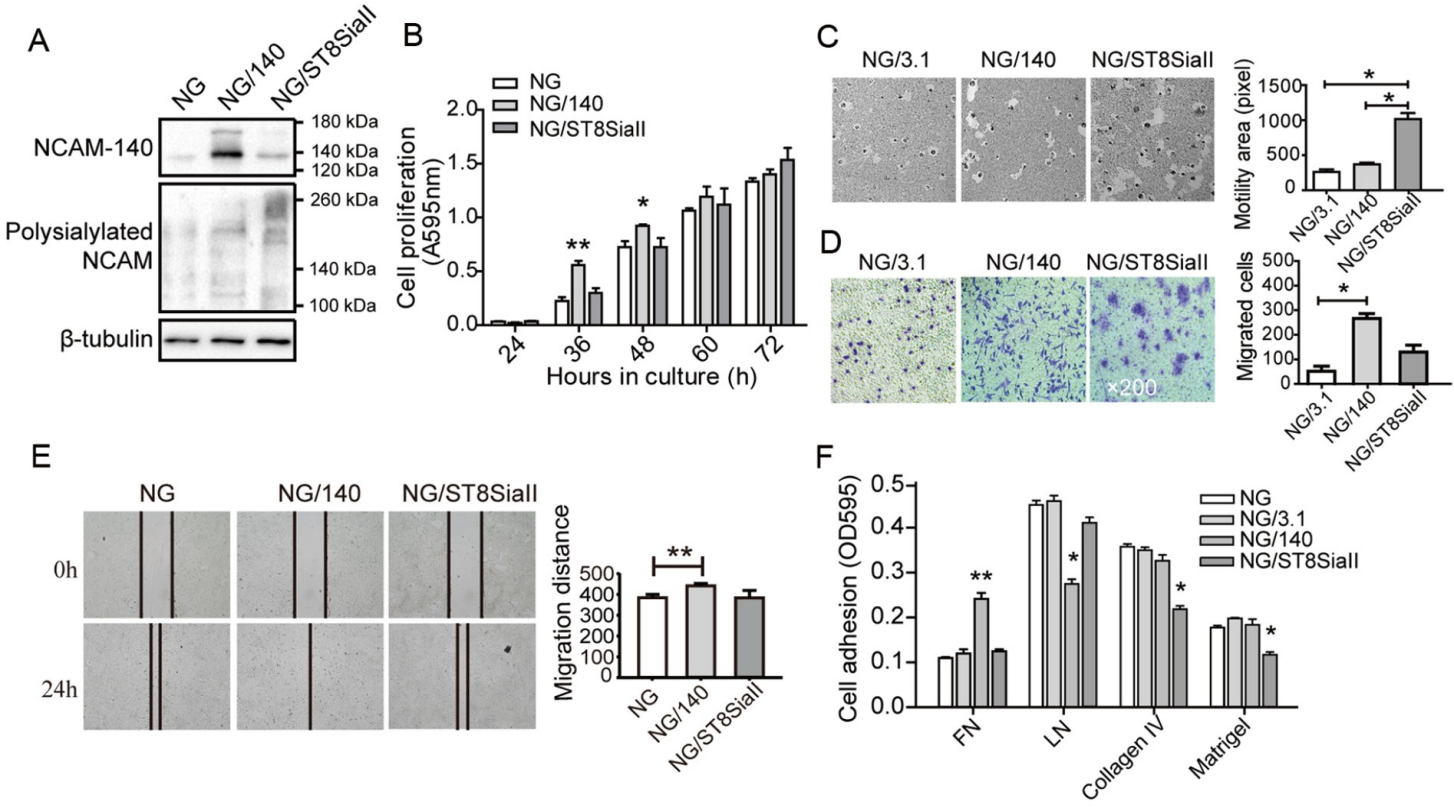
Figure 4 Differential effects of polysialylated NCAM and NCAM-140 on cell behaviors (A) Expressions of NCAM-140 and polysialylated NCAM in polySia-overexpressing cell lines detected by western blot analysis. (B) Proliferation assay. Transfected cells were cultured for 24, 36, 48, 60, or 72 h, and proliferation was assessed by MTT assay. (C) Motility assay, performed as described in Materials and Methods. Data are presentated as in Figure 3F. *P<0.05, **P<0.01. (D) Migration assay, and data are presented as in Figure 3D. *P<0.05. (E) Wound assay, performed as described in Materials and Methods. Confluent cells were wounded, incubated with 5 μg/mL mitomycin for 24 h, and wounds were photographed and marked using ImagePro Plus software. Results are presented as the average of migration distance (0‒24 h)±SD from three independent experiments. **P<0.01. (F) Adhesion assay. Transfected cells were cultured for 48 h, and cell adhesion to FN, laminin, collagen IV, Matrigel, and BSA solution was determined as described in Materials and Methods. Absorbance of crystal violet-stained cells was recorded at 595 nm. Four independent experiments gave similar results. *P<0.05, **P<0.01 vs NG/3.1.

We previously observed that polySia, catalyzed by ST8SiaII, facilitates NCAM-mediated cell migration in a polysialyltransferase-specific manner [22]. To assess the role of polySia in modulating NCAM-mediated cell behaviors, we cloned the ST8SiaII gene into NG, and the resulting cell lines were termed NG/ST8SiaII ( Supplementary Figure S2A,B). Western blot analysis results showed that polysialylated NCAM level were elevated in NG/ST8SiaII ( Figure 4A). Proliferation of NG/140 cells was significantly higher after 36 and 48 h of culture, and after 60 h NG/140 cells were almost completely confluent ( Figure 4B). These findings suggested that cell proliferation was modulated by NCAM-140 overexpression.

Cell motility was significantly increased in polySia-overexpressing NG/ST8SiaII cells but not in NG/140 cells ( Figure 4C), consistent with results shown in Figure 3F. Cell migration was increased in NG/140 cells but not in NG/ST8SiaII cells, indicating that this cell behavior was affected by NCAM-140, but not by polysialylation ( Figure 4D,E). Because NCAM is a type of adhesion molecule, the cell attachment to extracellular matrix (ECM) components (FN, laminin, collagen IV, Matrigel) ( Supplementary Figure S2C) was examined. NG/ST8SiaII cells showed reduced attachment to collagen IV and Matrigel, which is consistent with a previous study [31]. In contrast NG/140 cells showed reduced attachment to LN, increased attachment to FN and similar attachment to collagen IV compared to NG cells ( Figure 4F). Thus, polysialylated NCAM and NCAM-140 had different effects on cell adhesion to different ECM components.

### Polysialylated NCAM-mediated EGFR/STAT3 signaling pathway

**Figure 5:**
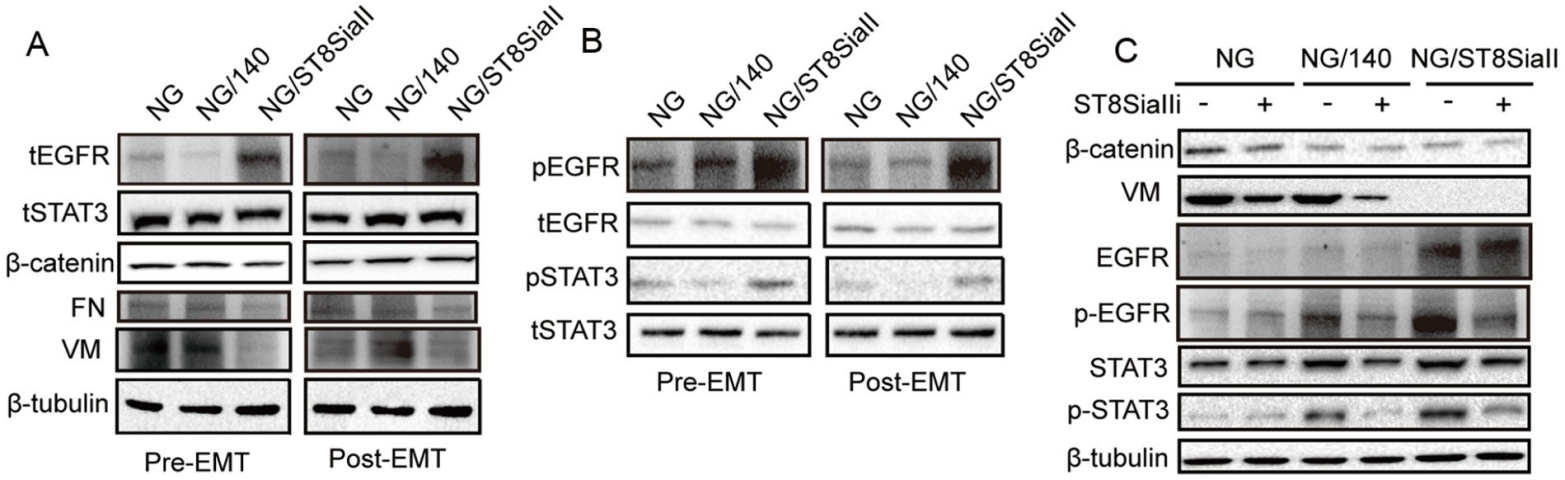
Figure 5 Polysialylated NCAM-mediated EGFR/STAT3 signaling pathway (A) Western blot analysis of transfected cells during EMT. tEGFR: total EGFR. tSTAT3: total STAT3. β-tubulin: loading control. (B) Western blot analysis of p-EGFR and p-STAT3. Equal amounts of tEGFR and tSTAT3 were subjected to western blotting, and p-EGFR (Tyr1068) and p-STAT3 (Tyr705) were detected. (C) Transfected or nontransfected NMuMG cells were further transfected with negative control RNA (-) or siRNA-targeting mouse ST8SiaII (+). Protein lysates were collected after 24 h and subjected to western blot analysis. β-tubulin: loading control.

The EGFR/STAT3 signaling pathway plays an important role in human BC [ 32, 33]. We examined the possible effects of polysialylated NCAM and NCAM-140 on this pathway. EGFR expression was significantly upregulated in NG/ST8SiaII cells compared with that in other NG cell lines. TGF-β1-induced EMT increased total EGFR (tEGFR) in NG/ST8SiaII cells but had no effect on β-catenin expression ( Figure 5A). EGFR and STAT3 phosphorylation were upregulated in NG/ST8Siall cells undergoing EMT ( Figure 5B), suggesting that the EGFR/STAT3 signaling pathway was activated by polysialylated NCAM but not by NCAM-140. When *polySia* was knocked down by silencing of *ST8SiaII* (ST8SiaIIi) ( Supplementary Figure S2D), phosphorylated EGFR and STAT3 levels were downregulated in all cell lines ( Figure 5C). These findings indicate that polySia is involved in the activation of the EGFR/STAT3 signaling pathway.

### NCAM-140 mediated β-catenin/slug signaling pathway

**Figure 6:**
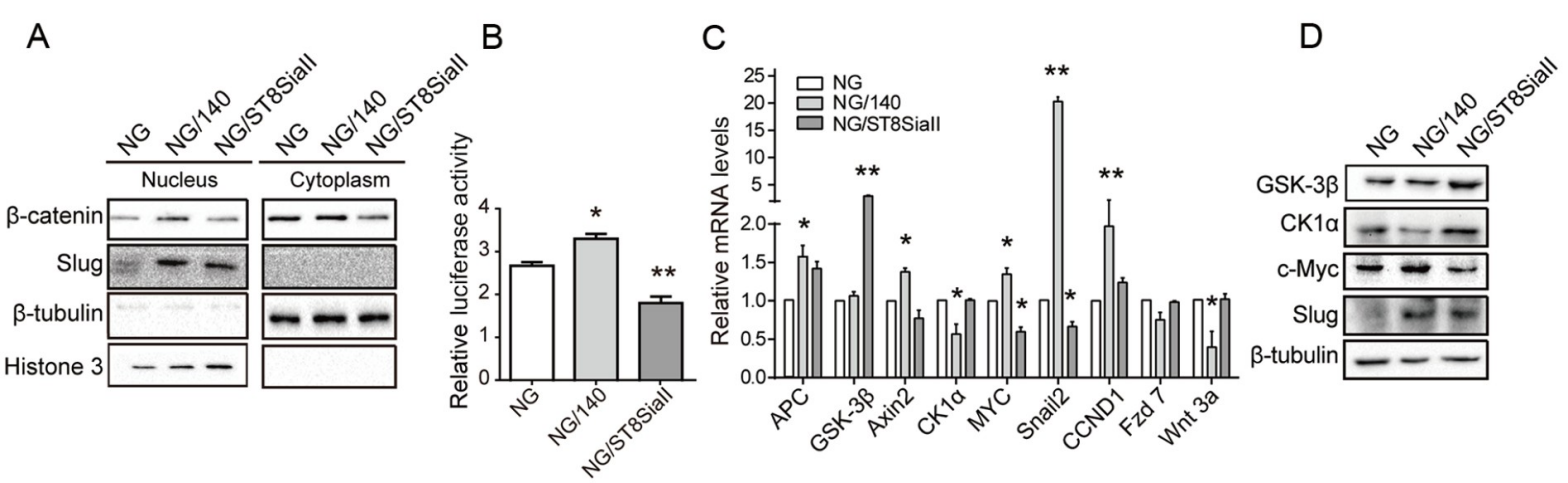
Figure 6 NCAM-140 mediates the β-catenin/slug signaling pathway (A) Western blot analysis. Lysates of transfected cells were fractionated as described in M&M, and subjected to SDS-PAGE. (B) Quantification of active β-catenin by luciferase gene reporter assay. Values of active β-catenin transcription were determined based on level of luciferase activity and normalized to internal control (Renilla luciferase plasmid). Data are shown as the mean±SEM from three independent experiments, expressed as relative activation in comparison with cells transfected with β-catenin-LEF/TCF insensitive (FOP) reporter vector. *P<0.05, **P<0.01. (C) RT-qPCR. *P<0.05, **P<0.01. (D) Western blot analysis. β-tubulin: loading control. Data are from three independent experiments and shown as the mean±SD.

Dissociation of E-cad/β-catenin complex is a key step in EMT, and alterations in localization and expression level of β-catenin have been observed in various types of cancers. The well-known EMT regulator/transcription factor slug has been shown to inhibit E-cad expression and promote cell metastasis [ 34, 35]. We found that β-catenin expression was increased in the NG/140 cell nuclei, but reduced in the NG/ST8SiaII cell nuclei ( Figure 6A). The finding suggests that NCAM-140 induces translocation of β-catenin from cytoplasm into the nucleus, and that such translocation is inhibited by polySia overexpression. Slug was accumulated in the nuclei of NG/140 cells. β-Catenin transcription was enhanced in NG/140 cells but reduced in NG/ST8SiaII cells ( Figure 6B). Expression of genes targeted by β-catenin (axin 2, c-myc, CCND1) was significantly upregulated in NG/140 cells ( Figure 6C).

In the absence of Wnt ligands, β-catenin is usually phosphorylated by GSK-3 at Ser33 and Ser37, leading to its ubiquitin-dependent degradation through incorporation of APC and CK1α [36]. We measured the mRNA and protein levels of GSK-3β and CK1α in our NG cell lines. The results showed that CK1α level was notably reduced in NG/140 cells, whereas GSK-3β was increased in polySia-overexpressing NG/ST8SiaII cells, suggesting that CK1α was downregulated in NG/140 cells to block β-catenin degradation, whereas GSK-3β was upregulated in NG/ST8SiaII cells to promote β-catenin degradation ( Figure 6C,D).

## Discussion

Modulation of tumor cell adhesion molecules is crucial in the control of the metastatic cascade. NCAM, the major substrate of polySia, has been associated with tumor invasion and the formation of metastatic deposits in many types of cancer [ 37, 38]. PolySia molecules greatly affect NCAM function, and are associated with malignant phenotype [ 11, 39]. Because of the large negative charge and repulsive characteristics of polySia, its presence inhibits the adhesive function of NCAM. We previously examined the role of polySia in NCAM function using the cell line ldlD-14. NCAM-140 strongly enhanced cell adhesion to FN and reduced adhesion to Matrigel, and these effects were reversed by the presence of polySia, indicating that polySia modulates NCAM-mediated cell behaviors [22]. In a recent study, ST8SiaII-overexpressing NIH-3T3 cells was found to exhibit reduced adhesion on Matrigel, while NCAM-overexpressing COS-7 cells showed increased adhesion to FN compared to ST8SiaII-overexpressing COS-7 cells [31], consistent with our findings ( Figure 4F). Given the similar changes in cell adhesion observed in NCAM-140 and ST8SiaII-overexpressing cells ( Figure 4D,E), FN may primarily stimulate cell migration. Our study also revealed the correlation of polySia expression with disease stage in BC patients, with high polySia expression in TGF-β1-treated NMuMG and MCF10A cells [28]. The roles of NCAM and polySia alterations in modulating various cell behaviors remain unknown.

EMT is a process whereby epithelial cells are transformed into cells with mesenchymal phenotypes, characterized by loss of cellular polarity and adhesion, and enhancement of invasiveness and migration. TGF-β1-induced EMT provides a useful *in vitro* model for studies of cancer cell responses to the tumor microenvironment. In the present study, we found that NCAM was overexpressed in clinical human BC tissues, and the expressions of NCAM-140 and polysialylated NCAM were greatly increased in MCF10A and NMuMG cells undergoing EMT. The most studied interaction partners in terms of NCAM function are FGF receptors (FGFR). Incubation of soluble NCAM with NCAM-negative cells promoted cell migration through the stabilization of FGFR1 and the sustained activation of Src [40]. Other functions of NCAM as a signaling receptor are independent of its interactions with FGF receptors. Lehembre *et al*. [41] showed previously that loss of E-cad function upregulated the expression of NCAM and promoted the translocation of NCAM into lipid rafts, consequently stimulated focal adhesion. Our study revealed that overexpression of NCAM-140 induced EMT in BC cells, and promoted cell migration through activation of β-catenin/slug signaling pathway. Considering the interaction between the Wnt/β-catenin and FGF pathway, the enhanced cell migration induced by NCAM-140 overexpression may be resulted from the combined effects of multiple signaling pathways [42], including the Wnt/β-catenin and FGF pathways.

We found that overexpression of NCAM-140 reduced E-cad expression, greatly enhanced FN expression, and promoted cell proliferation and migration. Although the Lehembre *et al*. [41] revealed the mechanistic links among loss of E-cad expression, NCAM function, focal adhesion assembly, and cell migration and invasion, they did not address the possible functional role of polySia in mediating the EMT process. We found that polySia overexpression caused a significant increase of cell motility but had a negligible effect on cell migration, indicating that NCAM, but not polySia, affects cell migration ability. Meanwhile, polysialylated NCAM and NCAM-140 have different effects on adhesion to various ECM components. In our previous work, polysialylation and polysialylated NCAM were both elevated under TGF-β treatment [28], overexpression of NCAM-140 significantly promoted cell proliferation, motility and migration in BC cells [22], which is consistent with the present study ( Figure 3C‒F). Cell migration was further enhanced by co-transfection of NCAM-140 and ST8SiaII in BC cells [22]. Accordingly, migratory ability was promoted by the overexpression of NCAM-140, but not by the overexpression of only ST8SiaII in the present study. These results indicated that NCAM-140 (but not polysialylation alone) plays an essential role in regulating cell migration.

Elevated levels of PSA-NCAM are linked to high-grade tumors characterized by undifferentiated cells and aggressive diffusion [43]. Because of polySia’s repulsive effect on polysialylated NCAM, the attachment of polySia onto NCAM inhibits its interaction with ECM components and receptors such as FGFR that regulate cell-cell and cell-ECM interactions [15]. In an FGFR independent manner, incubation with soluble NCAM or loss of polySia attenuates tumor cell migration and augments the number of focal adhesions by enhancing the association between p59Fyn with the focal adhesion scaffolding protein paxillin [44]. Our study demonstrated that polySia on NCAM modulates cell adhesion and promotes cell motility through activation of the EGFR/STAT3 pathway. Our findings suggest that polySia overexpression stimulates the EGFR/STAT3 signaling pathway. Given the fact that EGFR can form a complex with focal adhesion kinase [45], we hypothesized that focal adhesion kinase pathway might also be involved in the polySia-induced changes in cell adhesion and cell motility.

Mohadeseh *et al*. [46] revealed that the polysialylation of NCAM was enhanced during TGF-β1 induced EMT process, which is consistent with our findings ( Figure 2D). Previous study demonstrated that transcription of the *ST8SiaII* gene was perturbed by the downregulation of prion protein or β-catenin, which caused failure of generating NCAM polysialylation during EMT [46]. However, the effects of NCAM on β-catenin signaling have been less studied. In our study, the expression of β-catenin was unchanged when NCAM was overexpressed ( Figure 5A). NCAM-140 induces the translocation of β-catenin from cytoplasm to the nucleus, and promotes the β-catenin/slug signaling pathway. This translocation is inhibited by polySia overexpression, indicating that polysialylated NCAM and NCAM-140 affect cell behaviors through different signaling pathways. PolySia modification of NCAM appears to play a crucial role in its altered expression during EMT and its modulating function on cell behaviors.

**Figure 7:**
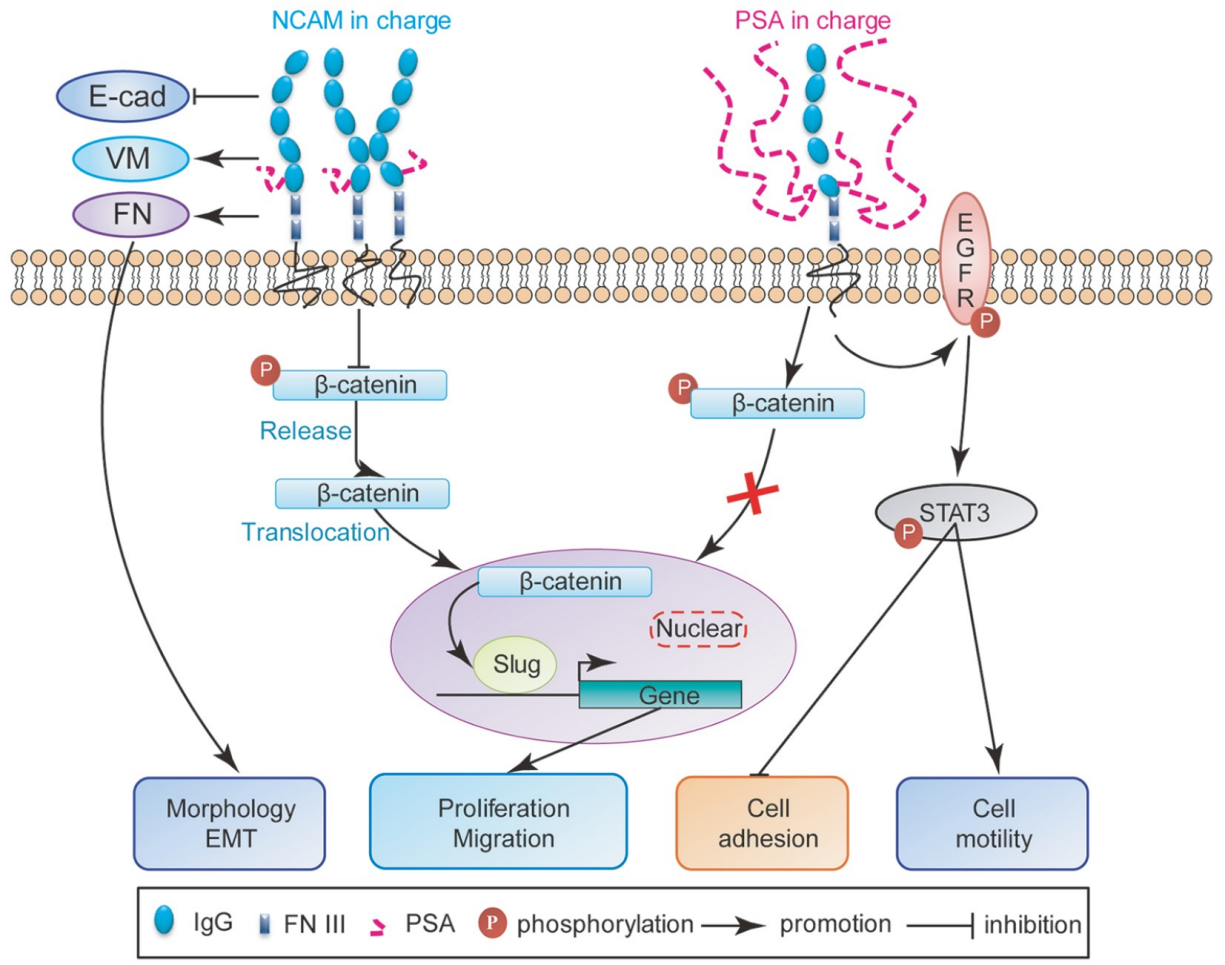
Figure 7 Possible transition pathway between NCAM-140-mediated β-catenin/slug signaling pathway and polysialylated NCAM-mediated EGFR/STAT3 signaling pathway

In summary, we demonstrate that NCAM-140 overexpression in NMuMG cells promotes translocation of released β-catenin into the nuclei, with consequent upregulation in the expressions of slug and β-catenin downstream genes, thereby facilitates cell proliferation and migration ( Figure 7). PolySia modification of NCAM stimulates the EGFR/STAT3 signaling pathway, inhibits cell adhesion to collagen IV and Matrigel, and increases cell motility ( Figure 7). The degree of polySia attachment to NCAM molecules is a crucial factor in modulating various pathways mediated by NCAM.

## Supporting information

Supplementary_materials

## Supplementary Data

Supplementary data is available at *Acta Biochimica et Biophysica Sinica* online.
