# Supplementary material for: NCAM and attached polysialic acid affect behaviors of breast epithelial cells through differential signaling pathways: Polysialylation in regulating NCAM function
Source: Acta Biochim Biophys Sin (Shanghai). 2024 Oct 15;56(11):1584–93. doi: 10.3724/abbs.2024176 (PMC11693866; doi:10.3724/abbs.2024176)
Supplement: Supplementary_materials [file Supplementary_materials.pdf]

Supplementary Table S1. Primers used in gene amplification

Gene names	Primer sequence	
	Sense (5′→3′)	Anti-sense (5′→3′)
Mouse *NCAM-180*	AGCCCCTTAGCAGCAGTAGCCG	CTGCCTCAGGTGGGTTCTTCACG
Mouse *NCAM-140*	GCTGACAGAACCCGAAAAGG	CCCATCATGCTTTGCTCTCAT
Mouse *NCAM-120*	GGCACCCGTCATTCTACCTA	GCAAAGGCTTGTCTTTGAGC
Human *NCAM*	TTGTTTTTCCTGGGAACTGC	TATCTTTGGCATCTCCTGCC
Mouse *ST8SiaII*	TCAGAATCAGAACCCCGTCA	CGACAGTCAGTTTCAATGCC
Mouse *ST8SiaIV*	ACTGAAAGTGCGAACTGCCT	GAGAAGACCTGTGCTGGGTC
Human *ST8SiaII*	TCAGAACCAGAACCCAGTCA	CGACAGTCAGTTTCAAAGCC
Human *ST8SiaIV*	ACTGAAAGTGCGAACTGCCT	GAGAAGACCTGTGCTGGGT
Mouse *c-Myc*	AGAGCTCCTCGAGCTGTTTG	ACGGAGTCGTAGTCGAGGTC
Mouse *Wnt3a*	ACTACGTGGAGATCATGCCC	GGTGGCTTTGTCCAGAACAG
Mouse *slug*	ATCTGTGGCAAGGCTTTCTC	GATGTGCCCTCAGGTTTGAT
Mouse *GSK-3β*	GCAGCCTTCAGCTTTTGGTA	AACTGACTTCCTGTGGCCTG
Mouse *Fzd7*	CCAGCGGTCAAGACAATCA	CCGATGAAGAGGTAGACGAACA
Mouse *cyclin D1*	AGACCTTTGTGGCCCTCTGT	GCAGGAGAGGAAGTTGTTGG
Mouse *CK1α*	CATCCCCAGTTGCTGTACG	GTCCCAGGAGATCCATGACT
Mouse *axin2*	TATGTCTTTGCACCAGCCAC	TGCATCTCTCTCTGGAGCTG
Mouse *APC*	GCCAGCTTTTACAGTCCCAG	GTGTTGCTTTCTGCCACTCC
Human *β-actin*	CTTCCTGGGCATGGAGTC	GCCGATCCACACGGAGTA
Mouse *γ-tubulin*	ATCTACCTGTCGGAGCATGG	GCCTCCCGATCTATGATGTC

Supplementary Table S2. **NCAM levels in normal and malignant breast tissue samples**

	Diagnosis	LN(P)	LN	Stage	D	NCAM (fd)
1	N	-	-	-	-	0.81±0.02
2	N	-	-	-	-	1.13±0.10
3	N	-	-	-	-	0.50±0.07
4	N	-	-	-	-	1.00±0.00
5	IDC	28	33	T2N3M0 IIIc	U	1.2±0.32
6	IDC	22	24	T1N3M0 IIIc	2	5.6±0.05
7	IDC	16	16	T2N3M0 IIIc	2.1	13.00±0.21
8	IDC	3	14	T4N1M0 IIIb	6	3.98±0.14
9	IDC	2	25	T4N1M0 IIIb	6.5	2.45±0.06
10	IC	U	U	T2N2M0 IIIa	3.4	7.11±0.43
11	SMC	1	28	T2N1M0 IIb	4	3.21±0.03
12	IDC	2	18	T2N1M0 IIb	2.3	1.51±0.02
13	ILC	3	22	T2N1M0 IIb	2.5	1.50±0.03
14	IDLC	0	23	T2N0M0 IIa	2.5	0.50±0.01
15	MC	0	19	T2N0M0 IIa	2.3	1.42±0.06
16	IDC-NS	1	25	T1N1M0 IIa	2	5.00±0.11
17	IDLC	1	24	T1N1M0 IIa	2	0.98±0.02
18	IDC	0	20	T2N0M0 IIa	2.8	0.81±0.03
19	IDC	0	12	T1N0M0 I	1.6	1.45±0.04
20	IDC	0	U	T1N0M0 I	1.2	1.04±0.00
21	IDC	0	U	T1N0M0 I	U	1.92±0.01
22	IDC	0	U	T1N0M0 I	1.0	1.10±0.01
23	IDC	0	U	T1N0M0 I	1.5	0.66±0.02
24	IDC	0	U	T1N0M0 I	U	0.74±0.05

LN(P), lymph node positivity; D, diameter (cm); fd, fold change; N, normal; IDC, infiltrating ductal carcinoma; IC, infiltrating carcinoma; SMC, squamous metaplastic carcinoma; ILC, infiltrating lobular carcinoma; IDLC, infiltrating ductal-lobular carcinoma; MC, mucinous carcinoma; IDC-NS, infiltrating ductal carcinoma with neural secretion; U, unknown. NCAM level was determined by quantitative RT-PCR and normalized relative to sample 4 (fold change) (see M&M). Values are shown as the mean±SEM.

Supplementary Table S3. Association of disease characteristics with NCAM gene expression in malignant BC patients

	Characteristics	TNM Stage			Total
		I	II	III	
No. of patients		6	8	6	20
Site:	Left	2	2	3	7
	Right	4	6	3	13
Histology	Invasive/Infil.	6	2	5	13
	Invasive/Infil.	0	1	0	1
	Invasive/Infil.	0	2	0	2
	Others	0	3	1	4
Lymph node involvement	Absent	6	3	0	9
	Present	0	5	6	11
	Unknown	0	0	0	0
NCAM positive		1/6 (17%)	4/8 (50%)	5/6 (83%)	10/20 (50%)

fd=fold change； fd>1.5=NCAM positive.

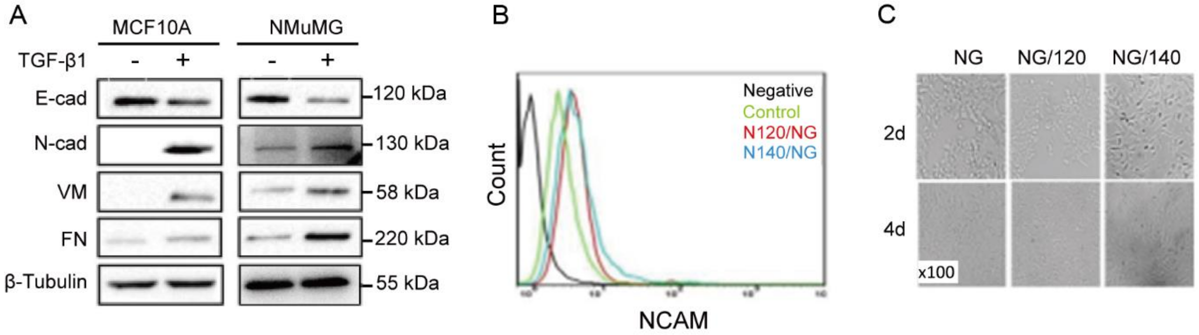

Supplementary Figure S1. **Verification of EMT cell model and NCAM overexpressing cell lines** (A) Western blot analysis of EMT markers. Aliquots from control cells or TGF-β1 treated cells were immunoblotted with specific antibodies. (B) Testing of NCAM overexpressing cell lines by flow cytometric analysis using anti-NCAM antibody. (C) Morphological changes induced by NCAM. Cells were cultured for 2 or 4 days and photos were taken. Magnification: 200×.

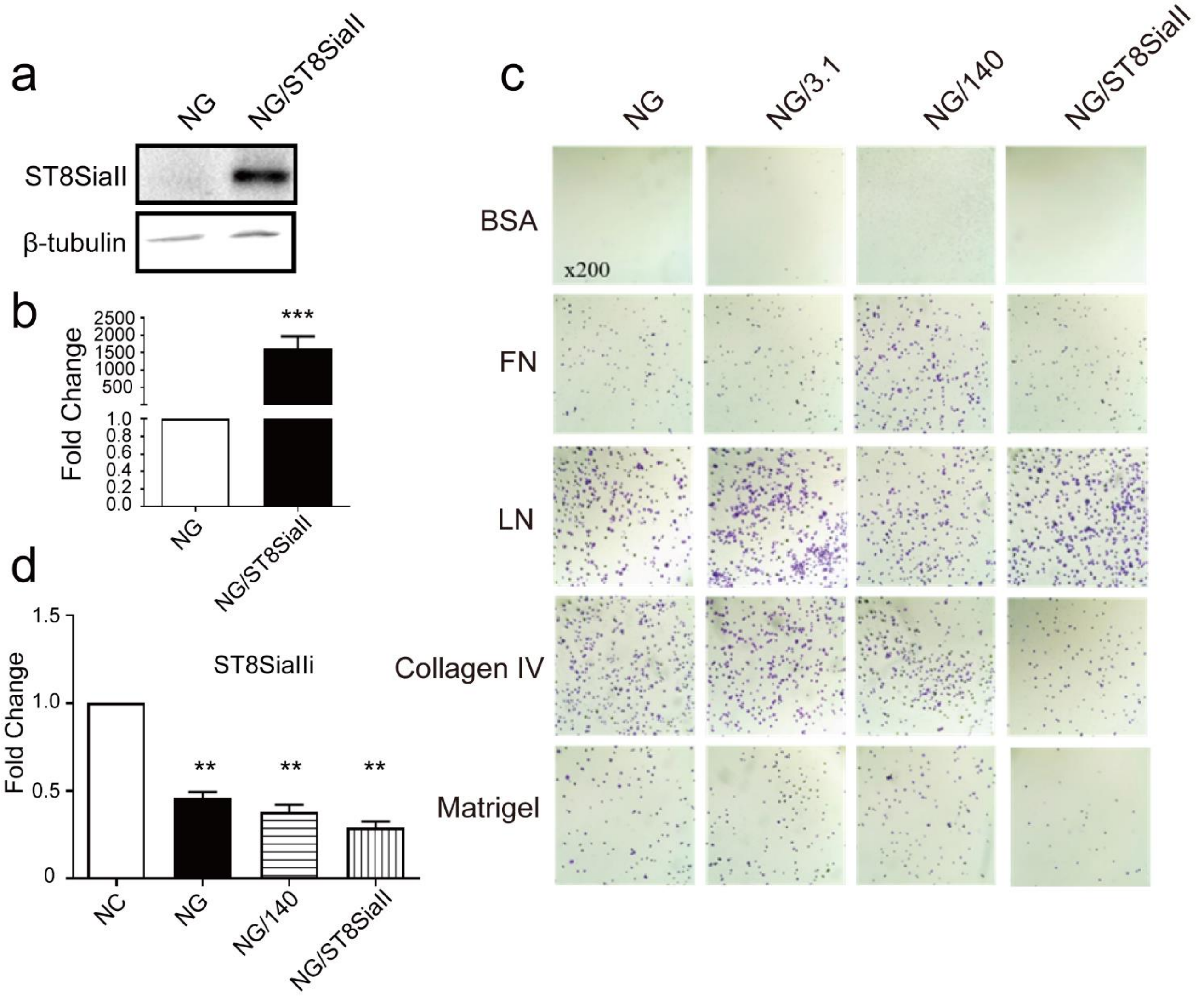

Supplementary Figure S2. **Verification of polysialylated NCAM in transfected cell lines and cell adhesion assay** (a) Western blot analysis of ST8SiaII expression in transfected cells using β-tubulin as the loading control. (b) mRNA levels of ST8SiaII genes in transfected cell lines were assessed by RT-qPCR, with *γ-tubulin* as the control. ***P<0.001. (c) Cell adhesion to FN, LN, collagen IV, matrigel or BSA solution was determined as described in M&M, and phase-contrast images were taken. (d) Down-regulation of ST8SiaII by siRNA. Cultured NMuMG and transfected cells were transfected with siRNA-targeting mouse ST8SiaII or with negative control RNA (NC). Quantitative RT-PCR was performed for ST8SiaII. **P<0.01.
